# Experimental and Computational Approaches to Sulfonated Poly(arylene ether sulfone) Synthesis Using Different Halogen Atoms at the Reactive Site

**DOI:** 10.3390/membranes12121286

**Published:** 2022-12-19

**Authors:** Seol Jang, Jung-Eun Cha, Seung Jae Moon, Justin Georg Albers, Min Ho Seo, Young-Woo Choi, Jong Hak Kim

**Affiliations:** 1 Fuel Cell Research and Demonstration Center, Future Energy Research Division, Korea Institute of Energy Research, Daejeon 56332, Republic of Korea; 2 Department of Chemical and Biomolecular Engineering, Yonsei University, 50 Yonseiro, Seodaemun-gu, Seoul 03722, Republic of Korea; 3 Fraunhofer Institute for Manufacturing Technology and Advanced Materials IFAM, Winterbergstrasse 28, 01277 Dresden, Germany; 4 Department of Nanotechnology Engineering, Pukyong National University, 45 Yongso-ro, Nam-gu, Busan 48547, Republic of Korea

**Keywords:** condensation polymerization, density functional theory, first-principle calculation, polymer electrolyte membrane, halogen atom

## Abstract

**Highlights:**

- The effect of differences in the halogen atoms at the ends of the biphenyl sulfone monomers on polymerization spontaneity was investigated through computer simulations and experiments.
- The polymerization process of SPAES was predicted using the first-principle calculation method.
- The simulation feasibility of polymer synthesis was verified experimentally.
- This approach based on quantum mechanics can even be effective in underlying polymer synthesis.

**Abstract:**

Engineering thermoplastics, such as poly(arylene ether sulfone), are more often synthesized using F-containing monomers rather than Cl-containing monomers because the F atom is considered more electronegative than Cl, leading to a better condensation polymerization reaction. In this study, the reaction’s spontaneity improved when Cl atoms were used compared to the case using F atoms. Specifically, sulfonated poly(arylene ether sulfone) was synthesized by reacting 4,4′-dihydroxybiphenyl with two types of biphenyl sulfone monomers containing Cl and F atoms. No significant difference was observed in the structural, elemental, and chemical properties of the two copolymers based on nuclear magnetic resonance spectroscopy, Fourier transform infrared spectroscopy, thermogravimetric analysis, X-ray diffraction, transmission electron microscopy, and electrochemical impedance spectroscopy. However, the solution viscosity and mechanical strength of the copolymer synthesized with the Cl-terminal monomers were slightly higher than those of the copolymer synthesized with the F-terminal monomers due to higher reaction spontaneity. The first-principle study was employed to elucidate the underlying mechanisms of these reactions.

## 1. Introduction

A fuel cell is an electrochemical device that can convert chemical energy into electrical energy, with high efficiency and low pollutant emissions. Among the different types of fuel cells, polymer electrolyte membrane fuel cells (PEMFCs), also known as proton-exchange-membrane fuel cells, are highly efficient power sources for transportation, stationary, and portable-energy applications. Over the last few decades, proton-exchange membranes (PEMs) have been widely developed as key materials and have received much attention because of their high proton conductivity, good mechanical strength, and good electrochemical performance [1,2,3]. Recently, using PEM has gained attention as an advantageous approach for high-purity hydrogen production using water electrolysis [4,5]. Various hydrocarbon-based alternatives have been explored despite the commercial availability of polytetrafluoroethylene (PTFE)-based polymers, such as Nafion, Flemion, Aciplex, and Dow series membranes because perfluorinated groups have diverse hazardous and detrimental effects on the environment [6,7,8].

The field of polymer chemistry has advanced significantly over the past few decades [9,10,11,12,13,14]; however, polymer synthesis is based on traditional material development methods that rely on the experience of researchers. To synthesize a polymer with the desired properties, it is necessary to find appropriate reactants, such as monomers, solvents, and reagents, and proceed with an appropriate synthetic methodology. However, the synthesis may not proceed appropriately, or a polymer with the desired properties may not be easily obtained. Moreover, the process might have to be repeated several times to obtain the desired polymer, thereby requiring a significant amount of time and costs.

Recently, many reports have been published on predicting chemical reactions using various methods [15,16,17,18,19,20,21,22,23,24,25,26]. Even in polymer science, efforts to predict polymer properties are still being explored, as published in various reports on computational approaches [27,28,29,30,31,32,33,34,35,36,37,38,39,40,41,42]. However, computational approaches do not easily predict and explain polymer synthesis because defining the representative chemical potential for polymers in DFT is difficult, with a high degree of freedom and computational complexity involving many atoms. Therefore, polymer synthesis is generally conducted in experimental approaches using general organic synthesis methods rather than using computational simulations. The polymer-synthesis prediction process can proceed more rapidly if performed without an experience-based method, which has to follow costs and human resources, thereby compensating for polymer synthesis’s time and economic disadvantages. Density functional theory (DFT) based on quantum mechanics [43,44] can be chemically simulated to thermodynamically investigate the polymerization process. Predicting the spontaneity of reactions through thermodynamic analysis is one of the promising solutions used to improve existing methods.

F-terminal biphenyl sulfone has mainly been used in the synthesis of sulfonated poly(arylene ether sulfone) (SPAES), probably because the F atom is considered more electronegative than Cl, leading to a better reaction [45,46,47,48,49,50]; however, no clear explanation or mechanism has been reported. Since the polymerization proceeds stepwise, it was considered that the difference in the halogen atoms of the monomers would have a significant effect on the overall polymerization process, and consequently, there would be a difference in the reaction yield [51,52,53]. Therefore, in this study, the effect of differences in the halogen atoms at the ends of the biphenyl sulfone monomers on polymerization spontaneity was investigated in detail through computer simulations and experiments. Specifically, the polymerization process of SPAES used as a hydrocarbon-based PEM for fuel cells was predicted using the first-principle calculation method, and the simulation feasibility of polymer synthesis was verified experimentally. Cl and F were used as terminal atoms in the diphenylsulfone-based monomers. The structural, elemental, and physicochemical properties of the two synthesized SPAES copolymers were characterized using various techniques, such as nuclear magnetic resonance (NMR), Fourier transform infrared (FTIR) spectroscopy, thermogravimetric analysis (TGA), X-ray diffraction (XRD), transmission electron microscopy (TEM), and electrochemical impedance spectroscopy (EIS), as well as employing a universal tensile machine (UTM) and viscometer.

## 2. Materials and Methods

### 2.1. Computational Details

The polymerization process was simulated using quantum-mechanics-based DFT calculations. The Vienna ab initio simulation package (VASP) was used for DFT calculations [54]. The chemical structures of all the compounds participating in the synthesis were constructed using the Materials Studio software (BIOVIA, Waltham, MA, USA). The exchange-correlation energies of electrons were described using the revised Perdew–Burke–Ernzerhof (RPBE) functional for generalized gradient approximation (GGA) [55,56]. The geometry optimizations were conducted until the energy was converged to less than 1 × 10^−4^ eV using a plane wave kinetic energy cutoff of 520 eV. Monkhorst-pack k-point meshes were used in 1 × 1 × 1 and 6 × 1 × 1 in gas and polymer, respectively. In addition, the calculations converged upon the force threshold of 1 × 10^−3^ eV Å^−1^. For the structural relaxations, the Brillouin zone was integrated using the Methfessel–Paxton smearing method [57] with a smearing width of 0.01 eV.

### 2.2. Materials

4,4′-Dihydroxybiphenyl (BP), bis(4-chlorophenyl) sulfone (DCDPS), potassium carbonate, toluene, and *N*,*N*-dimethylacetamide (DMAc) were purchased(Merck, Rahway, NY, USA). Bis(4-fluorophenyl) sulfone (DFDPS), bis(4-chlorophenyl-3-sulfophenyl) sulfone disodium salt (SDCDPS), and bis(4-fluorophenyl-3-sulfophenyl) sulfone disodium salt (SDFDPS) were purchased(Yanjin, China). All materials were of analytical grade and used without further purification.

### 2.3. Synthesis of the SPAES Copolymer

SPAES was synthesized via condensation polymerization. DCDPS (4.307 g, 15 mmol), SDCDPS (7.368 g, 15 mmol), BP (5.586 g, 30 mmol), potassium carbonate (9.121 g, 66 mmol), and DMAc (40 mL) was added to a 3-neck flask equipped with a Dean-Stark trap, nitrogen inlet, and condenser. Toluene (60 mL) was added to the above reaction mixture for water removal. Further, the reaction mixture was heated at 135 °C for 6 h and refluxed under continuous stirring. Subsequently, toluene was distilled off, and the temperature was increased to 165 °C and maintained for 18 h. Afterward, the reaction mixture was cooled, diluted with DMAc, and poured into distilled water. The precipitated copolymer was washed with distilled water and dried in an oven at 120 °C for 24 h. Synthesis using DFDPS and SDFDPS monomers was performed via the same method.

### 2.4. Membrane Preparation

Free-standing membranes with a thickness of approximately 40 μm were prepared using the solution-casting method. Each synthesized SPAES copolymer was dissolved in DMAC to prepare a 10 wt% solution. After the SPAES copolymer dissolved completely, the solution was poured into a Petri dish, followed by drying in a drying and vacuum oven at 120 °C for more than 6 h. The dried SPAES membrane was converted into the proton form by immersion in 2 M HCl at room temperature for 24 h, and it was then washed with deionized water, followed by drying in a vacuum oven at 80 °C.

### 2.5. Characterization of the SPAES Copolymer

The successful synthesis of the SPAES copolymer was confirmed using NMR and FTIR spectroscopy. ^1^H NMR measurements were conducted on a JNM-ECZ500R (JEOL, Tokyo, Japan) 500 MHz spectrometer with polymer solutions in DMSO-*d_6_*. The FTIR spectra of the SPAES copolymer were measured using ALPHA2 (Bruker, Billerica, MA, USA) after preparing the membrane. TGA was conducted using an N-1000 system (Scinco, Seoul, Korea) from 25 to 800 °C at a heating rate of 10 °C/min under a nitrogen atmosphere to investigate the thermal stability of the SPAES copolymer. The viscosity of the SPAES copolymer was measured using a DV2T (Brookfield) viscometer, using a solution mixture of SPAES in DMAC. The morphology of the SPAES copolymer was observed via TEM (JEM-F200, JEOL, Japan). The crystalline structures of the copolymers and electrolytes were characterized using XRD (SmartLab, Rigaku, Japan). The mechanical properties of the copolymer membranes were obtained using a UTM (MultiTest1-i, Mecmesin) at a crosshead speed of 30 mm min^−1^.

To measure the ion-exchange capacity (IEC), the SPAES membranes (3 cm × 3 cm) in proton form were immersed in 40 mL of 3 M NaCl aqueous solution for 24 h and titrated with 0.01 M NaOH solution using an electronic titrator (Metrohm 848 Titrino Plus), and IEC values were calculated as follows:

(1) $$\mathrm{IEC}\left(\frac{\mathrm{meq}}{g}\right)=\frac{V_{NaOH}C_{NaOH}}{M_{dry}}$$

where *V_NaOH_* is the volume of the NaOH solution consumed (mL), *C_NaOH_* is the molar concentration of the NaOH solution (M), and *M_dry_* is the mass of the dried membrane (g).

To measure the proton conductivity, the SPAES membranes (3 cm × 3 cm) in proton form were immersed in deionized water at room temperature for more than 2 h and evaluated using an electrochemical-impedance spectrometer (HCP-803 potentiostat, Biologic).

(2) $$\sigma \left(\frac{S}{\mathrm{cm}}\right)=\frac{L}{RWd}$$

where *σ* is the proton conductivity, *L* is the distance between the working and counter platinum wire electrodes, *R* is the measured membrane resistance, *W* is the membrane width, and *d* is the membrane thickness.

## 3. Results and Discussion

### 3.1. Synthesis and Characterization of the SPAES Copolymer

**Figure 1:**
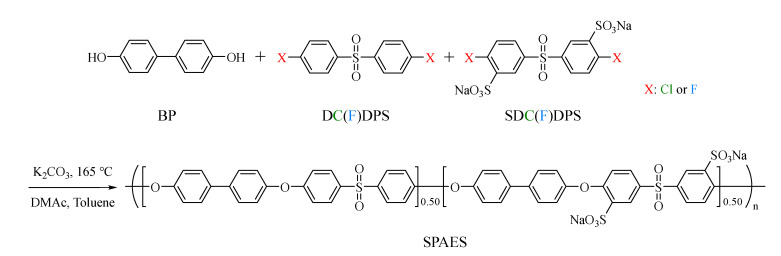
Synthesis scheme for the SPAES copolymer.

**Figure 2:**
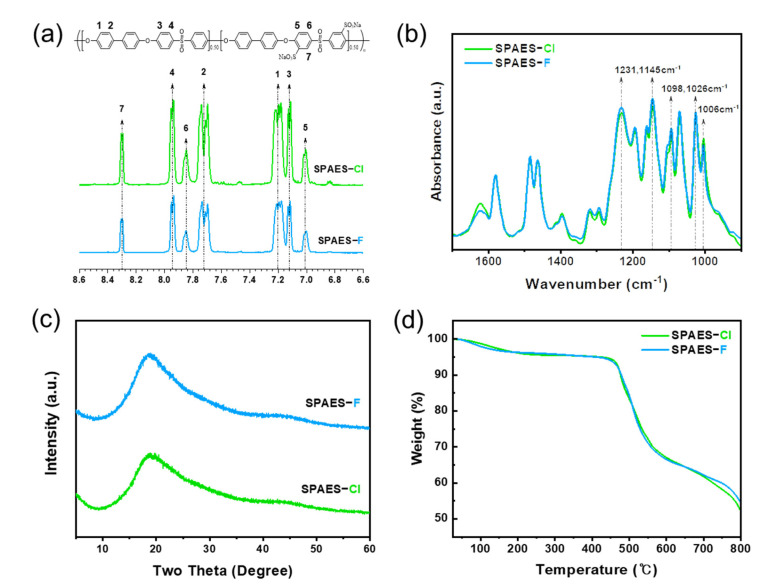
(**a**) ^1^H NMR and (**b**) FTIR spectra, (**c**) XRD patterns, and (**d**) TGA curves of SPAES copolymers.

To clearly and systematically investigate the effect of halogen atoms on polymer synthesis, two types of SPAES were synthesized via condensation polymerization using Cl- and F-terminal biphenyl sulfone monomers, as shown in Figure 1. The polymers synthesized with the Cl- and F-terminal monomers were named SPAES-Cl and SPAES-F, respectively. First, the chemical structures and successful synthesis of the SPAES copolymers were confirmed using ^1^H NMR and FTIR spectroscopic techniques. The target degree of sulfonation (DS) for SPAES was 50; however, a slightly lower DS (approximately 48) was obtained, as characterized via NMR spectroscopy (Figure 2a). When the feed monomer ratio of DC(F)DPS and SDC(F)DPS was 1:1, the DS value was reduced due to the difference in the chemical structure [58,59]. ^1^H NMR (DMSO-d6, 400 MHz): δ 8.30 (br, 2H, ArH), 7.96 (br, 4H, ArH), 7.87 (br, 2H, ArH), 7.73 (br, 8H, ArH), 7.21 (br, 7.95H, ArH), 7.13 (br, 4H, ArH), and 7.02 (br, 2H, ArH). No significant difference was observed between the FTIR absorption spectra of the two copolymers, as shown in Figure 2b. The strong absorption bands at 1098 and 1026 cm^−1^ were assigned to the characteristic symmetric and asymmetric stretching vibrations of sulfate groups [60]. The aromatic sulfoxide group was observed at 1231 cm^−1^ and 1145 cm^−1^ [61], while the diphenyl ether band appeared at 1006 cm^−1^, indicating the absence of residual monomers in the final products. ^1^H NMR and FTIR spectroscopic results showed that the synthesis of both SPAES-Cl and SPAES-F copolymers was successful, and no significant differences were observed in their molecular structure and chemical components.

The crystallinity and structural properties of the SPAES copolymers were characterized via XRD patterns, as shown in Figure 2c, where the intensity of X-ray scattering is plotted against the diffraction angle, 2θ. Both the SPAES-Cl and SPAES-F copolymers did not show any sharp, narrow peaks, indicating a lack of crystallinity and a completely amorphous nature of the copolymer. The amorphous nature of SPAES copolymers is due to the random arrangement of the monomeric units, leading to the perturbation of the long-range order between the chains [46,62]. The *d*-spacing was calculated using Bragg’s relation: *nλ* = 2dsin(θ), where *n* is an integer (1), *λ* is the wavelength of the incident light (1.54 Å), and *d* is the average inter/intrachain distance of the copolymer. The maximum intensity peak was observed at 18.8°, and therefore, the *d*-spacing was determined to be approximately 4.7 Å for both copolymers.

Figure 2d shows the TGA curves of the copolymers in the Na^+^ form. Less than 5% weight loss occurred initially up to ~420 °C due to the removal of bound water. Thereafter, as the polymer main chain decomposed, a significant amount of weight loss occurred. SPAES-Cl and SPAES-F copolymers showed similar degradation patterns, indicating that their thermal stability and properties were not significantly different.

**Figure 3:**
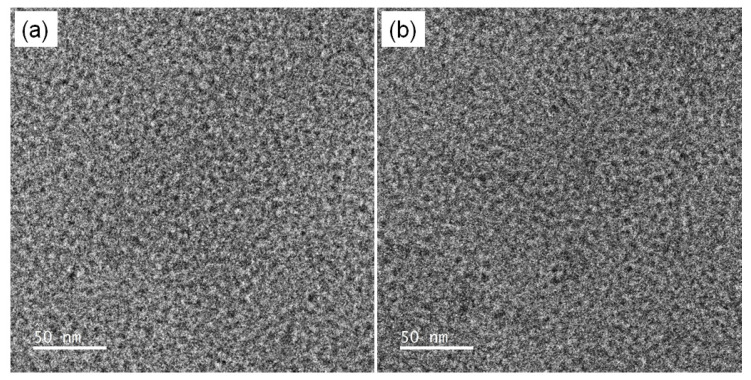
TEM images of SPAES copolymers; (**a**) SPAES-Cl and (**b**) SPAES-F.

TEM analysis was performed to characterize the morphological properties of the SPAES copolymers. Figure 3 shows TEM images of the unstained SPAES-Cl and SPAES-F copolymers. The dark regions represent the localization of ionic SO_3_H domains due to the sulfonic acid groups, whereas the bright regions represent the non-ionic-conducting domains due to the higher electron density of the former. Both the SPAES-Cl and SPAES-F copolymers exhibited well-defined microphase-separated morphologies, providing effective ionic channels with a nanometer-scale distribution throughout the membranes. This morphology was responsible for obtaining high proton conductivity without losing the mechanical strength of the membranes. No significant difference was observed in the nanometer-scale morphological properties between the SPAES-Cl and SPAES-F copolymers. Similarly, the IEC values and proton conductivities of the two copolymers were not significantly different, as listed in Table 1.

**Figure 4:**
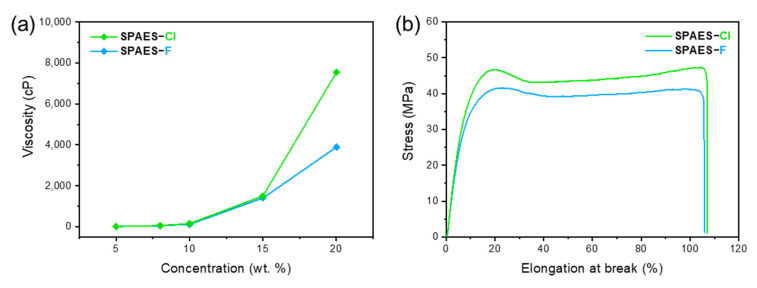
(**a**) Solution viscosity and (**b**) stress–strain curves of SPAES copolymer membranes.

The chemical properties of the SPAES-Cl and SPAES-F copolymers did not differ significantly, as evident from characterization results using various techniques, and therefore, the physical properties, such as the mechanical strength and viscosity, of the copolymers were measured. First, the viscosities of the two copolymers in DMAc were measured as a function of the polymer concentration, as shown in Figure 4a. The results show that the viscosity of the SPAES-Cl copolymer was always higher than that of SPAES-F, and the difference between them increased with increasing concentration. This indicates that higher-molecular-weight SPAES copolymers were produced when using Cl-terminal monomers compared to those obtained using F-terminal monomers for condensation polymerization.

Good mechanical properties are crucial for the widespread application of PEM; therefore, the tensile stress–strain curves of SPAES copolymer membranes were measured using a UTM, as shown in Figure 4b. Both the SPAES copolymer membranes exhibited mechanical properties that were sufficiently high for various applications. Specifically, the tensile stress at the yield of the SPAES-Cl copolymer membrane was 47 MPa, while the elongation at break reached 108%. When comparing the two copolymers, the SPAES-Cl copolymer membrane showed better tensile properties than the SPAES-F membrane, which was due to the higher molecular weight, as characterized by solution viscosity measurements. This is because polymers with higher molecular weights have higher mechanical strength due to increased intermolecular attractive forces [63,64,65].

### 3.2. DFT Calculations

**Figure 5:**
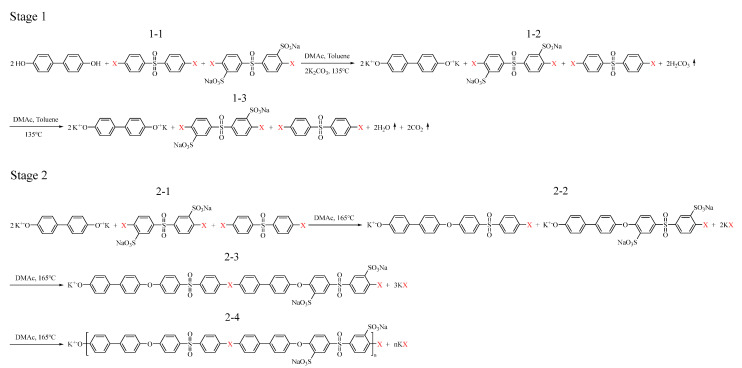
Synthesis steps for SPAES polymerization.

The SPAES synthesis process was divided into stages 1 and 2, as shown in Figure 5. In stage 1, the functional group of BP was substituted from -OH to -OK (when the functional groups of BP are -OH and -OK, they are referred to as BP (-OH) and BP (-OK), respectively), which were ready to react with DC(F)DPS or SDC(F)DPS. In stage 2, BP was bonded to DC(F)DPS and SDC(F)DPS to form the SPAES polymer. Therefore, the difference in spontaneity between SPAES-Cl and SPAES-F was obtained in stage 2. Periodic boundary conditions, assuming infinite chains, were applied to simulate SPAES polymers composed of multiple repeating units (Figures S15 and S16).

Structural optimization using DFT calculations was performed for all the compounds participating in synthesizing the SPAES copolymer (Figure 6 and Figures S3–S16). The likelihood of a structure existing in nature can be predicted by calculating the chemical structure with the lowest DFT energy’s simulating chemical potential. Gibbs free energy changes of each structure were calculated according to Equation (3) [66].

ΔG = ΔE + ΔZPE − TΔS(3)

where G is the Gibbs free energy, E is DFT energy, ZPE is zero-point energy, T is the temperature, and S is the entropy. In the calculation, the phonon contribution was not considered, and the polymer and salts were assumed to be solid-state.

**Figure 7:**
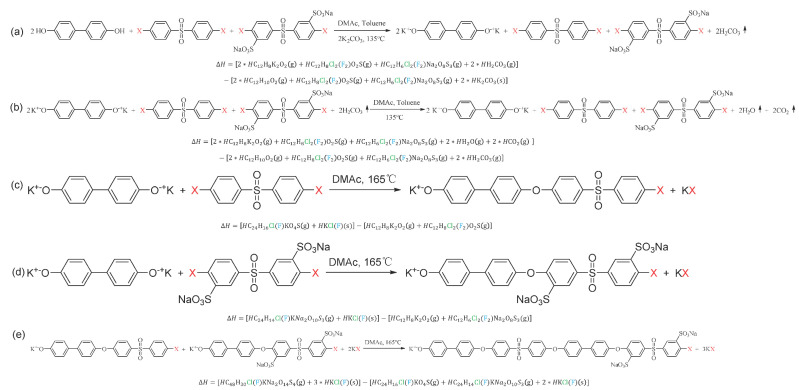
Each synthesis step and the equation of enthalpy change (Δ*H*): (**a**) BP(-OH form) is converted to BP(-OK form); (**b**) carbonic acid is converted to water and carbon dioxide; (**c**) BP(-OK) and DC(F)DPS are bonded to form a hydrophobic domain; (**d**) BP(-OK) and SDC(F)DPS are bonded to form a hydrophilic domain; (**e**) hydrophobic and hydrophilic domains are bonded to form a single SPAES unit.

The Gibbs free energy change can be obtained by calculating the chemical potential for the SPAES polymer and salts since it was assumed to be in a solid phase minimizing the entropy effect. The chemical potential was obtained using the equation shown in Figure 7 for each step. Given this, salts were assumed to be in a solid state, and other compounds were assumed to be in a gaseous state. To find a stable structure, we tried to calculate by considering the possible structures with flat and distorted models (Figures S1 and S2).

### 3.3. Gibbs Free Energy Diagram

**Figure 8:**
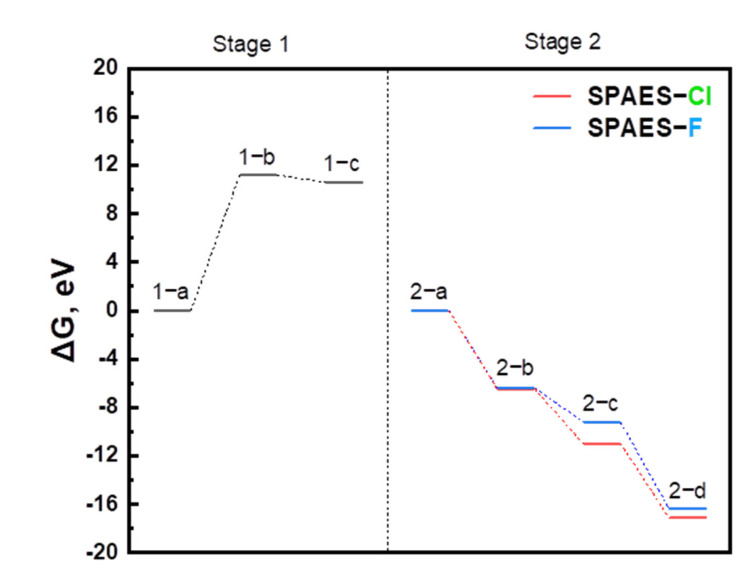
Gibbs free energy (Δ*G*) for the SPAES synthesis reaction. In stage 1, BP(-OH) is substituted with BP(-OK), and in stage 2, the substituted BP(-OK) is reacted with DC(F)DPS or SDC(F)PDS with a halogen group.

Figure 8 shows the Gibbs free energy diagram obtained from these calculations. The Gibbs free energy increased from steps 1-a to 1-b. In the polycondensation reaction in which water was removed, the -OH form was changed to the -OK form and an unstable state that easily reacted with a halogen element. In steps 1-b to 1-c, carbonic acid was rapidly converted into water and carbon dioxide. The difference in spontaneity between the SPAES-Cl and SPAES-F copolymers can be observed in stage 2.

The Gibbs free energy diagram shows that the reaction’s spontaneity was more favorable for SPAES-Cl, presenting thermodynamic stability, than those of SPAES-F. In stage 2-c, the difference in Gibbs free energy between the SPAES-Cl and SPAES-F copolymers was noticeable, and the difference decreased slightly in stage 2-d. This implied that fluorine atoms, which are more electronegative than chlorine atoms, maintain their original structure more effectively because of the strong bonding between fluorine and carbon atoms. This simulation result shows that the calculations are considerably simplified in that the high degree of freedom of the polymer geometrical configuration and number of atoms is limited, but more stable polymer polymerization from Cl halogen atoms enables the stable preparation of modified SPES polymerizations of various structures. Accordingly, these approaches based on quantum mechanics can even be effective at underlying polymer synthesis.

## 4. Conclusions

Two types of SPAES copolymers were synthesized via condensation polymerization using two types of biphenyl sulfone monomers containing Cl and F atoms. The solution viscosity and mechanical strength of the copolymer synthesized with the Cl-terminal monomers were higher than those synthesized with the F-terminal monomers, although no significant differences were observed in the structural, elemental, and chemical properties of the two copolymers. In DFT simulation, the thermodynamic formation of SPAES-Cl was more favorable than those of SPAES-F. This formation can cause a higher molecular weight of SPAES-Cl, enhancing mechanical strength and solution viscosity. Based on both approaches, a computational approach is a good option for predicting experimental synthesis with accuracy and has shown to be an effective material-development method.

## Figures and Tables

**Figure 6:**
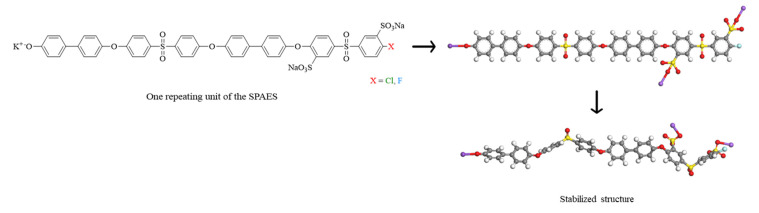
Structural optimization process of one repeating unit of the SPAES copolymer.

**Table 1:** IEC and proton conductivity of SPAES copolymer membranes.

Polymer	IEC (mmol∙g^−1^) ^1^		Proton Conductivity (S∙cm^−1^) ^2^
	Theoretical	Experimental	
SPAES-Cl	2.08	1.82	0.119
SPAES-F		1.81	0.124

^1^ The theoretical IEC was calculated from the ^1^H NMR results, and the experimental IEC values were measured for samples immersed in 40 mL of 3 M NaCl aqueous solution for 24 h and titrated with 0.01 M NaOH solution using an electronic titrator. ^2^ Proton conductivities were measured using an electrochemical-impedance spectrometer under full hydration at 25 ℃.

## Data Availability

Data available on request due to restrictions eg privacy or ethical. The data presented in this study are available on request from the corresponding author.

## Supplementary Materials

The following supporting information can be downloaded at: https://www.mdpi.com/article/10.3390/membranes12121286/s1, Figure S1. A repeating unit of SPAES calculated by applying COMPASSII, flat, and rotation respectively. Figure S2. Illustration of rotating one side of a symmetric phenyl group. Figure S3. 4,4′-Dihydroxybiphenyl (BP, -OH form). Figure S4. 4,4′-Dihydroxybiphenyl (BP, -OK form). Figure S5. Bis(4-chlorophenyl) sulfone. Figure S6. Bis(4-fluorophenyl) sulfone. Figure S7. Bis(4-chlorophenyl-3-sulfophenyl) sulfone disodium salt. Figure S8. Bis(4-fluorophenyl-3-sulfophenyl) sulfone disodium salt. Figure S9. Bonding of 4,4′-Dihydroxybiphenyl and bis(4-chlorophenyl) sulfone. Figure S10. Bonding of 4,4′-Dihydroxybiphenyl and bis(4-fluorophenyl) sulfone. Figure S11. Bonding of 4,4′-Dihydroxybiphenyl and bis(4-chlorophenyl-3-sulfophenyl) sulfone disodium salt. Figure S12. Bonding of 4,4′-Dihydroxybiphenyl and bis(4-fluorophenyl-3-sulfophenyl) sulfone disodium salt. Figure S13. Sulfonated poly(arylene ether sulfone)(Cl form). Figure S14. Sulfonated poly(arylene ether sulfone)(F form). Figure S15. Sulfonated poly(arylene ether sulfone)(Cl form, periodic boundary condition). Figure S16. Sulfonated poly(arylene ether sulfone)(F form, periodic boundary condition). Figure S17. Gibbs free energy (ΔG) difference depending on the position of monomers and the state of salt.

## Abbreviations

DFT: density functional theory
BP: 4,4′-dihydroxy biphenyl
DCDPS: bis(4-chlorophenyl) sulfone
DFDPS: bis(4-fluorophenyl) sulfone
SDCDPS: bis(4-chlorophenyl-3-sulfophenyl) sulfone disodium salt
SDFDPS: bis(4-fluorophenyl-3-sulfophenyl) sulfone disodium salt
SPAES: sulfonated poly(arylene ether sulfone)
VASP: Vienna ab initio simulation package
RPBE: revised Perdew–Burke–Ernzerhof
GGA: generalized gradient approximation
