# Supplementary material for: Experimental and Computational Approaches to Sulfonated Poly(arylene ether sulfone) Synthesis Using Different Halogen Atoms at the Reactive Site
Source: Membranes (Basel). 2022 Dec 19;12(12):1286. doi: 10.3390/membranes12121286 (PMC9785268; doi:10.3390/membranes12121286)
Supplement: Supplementary file 1 [file membranes-12-01286-s001.zip › membranes-2086034-supplementary.pdf]

Supporting information

Experimental and Computational Approaches to Sulfonated Poly(Arylene Ether Sulfone) Synthesis Using Different Halogen Atoms at the Reactive Site

Seol Jang[1,2], Jung-Eun Cha[1], Seung Jae Moon[2], Justin Georg Albers[3], Min Ho Seo[4,*], Young-Woo Choi[1,*], Jong Hak Kim[2,*]

[1] *Hydrogen Energy Research Division, Korea Institute of Energy Research, 152 Gajeong-ro, Yuseong-gu, Daejeon 34129, Republic of Korea*

[2] *Department of Chemical and Biomolecular Engineering, Yonsei University, 50 Yonseiro, Seodaemun-gu, Seoul 03722, Republic of Korea*

[3] *Fraunhofer Institute for Manufacturing Technology and Advanced Materials IFAM, Winterbergstrasse 28, 01277 Dresden, Germany*

[4] *Department of Nanotechnology Engineering, Pukyong National University, 45 Yongso-ro, Nam-gu, Busan 48547, Republic of Korea*

* Corresponding author

E-mail address: cozmoz67@kier.re.kr

E-mail address: foifrit@pknu.ac.kr

E-mail address: jonghak@yonsei.ac.kr

Polymer synthesis is a traditional method of material development that depends on researcher's experience. Based on quantum mechanics, DFT; density functional theory can be chemically simulated through the computational simulations to investigate the polymerization process thermodynamically. Therefore, if the spontaneity of a reaction can be predicted in advance without an experimental approach, it is expected that the existing traditional method can be improved. Thermodynamic analysis was conducted through the change of Gibbs free energy and derived from the thermodynamic internal energy obtained through geometric optimization of DFT calculations.

S1. Geometric optimization

Structural optimization using DFT calculations was performed for all the compounds participating in the synthesis of the SPAES copolymer (**Figures S3-S16**). We intended to design to simulate structures like those existing in nature. As a result of geometrical optimization of the structure to which the COMPASSII forcefield was applied, it showed remarkably excessive torsion. So, in order to derive the result only through DFT calculation, we tried to calculate with a flat structure without applying COM2. However, the structure was far from the actual structure, and was eventually calculated by rotating one of the two phenyls by 30 degrees. (**Figure S2**). The stable structures obtained through this method were bonded to each other and geometric optimization was performed in the same way until a single SPAES polymer was formed.

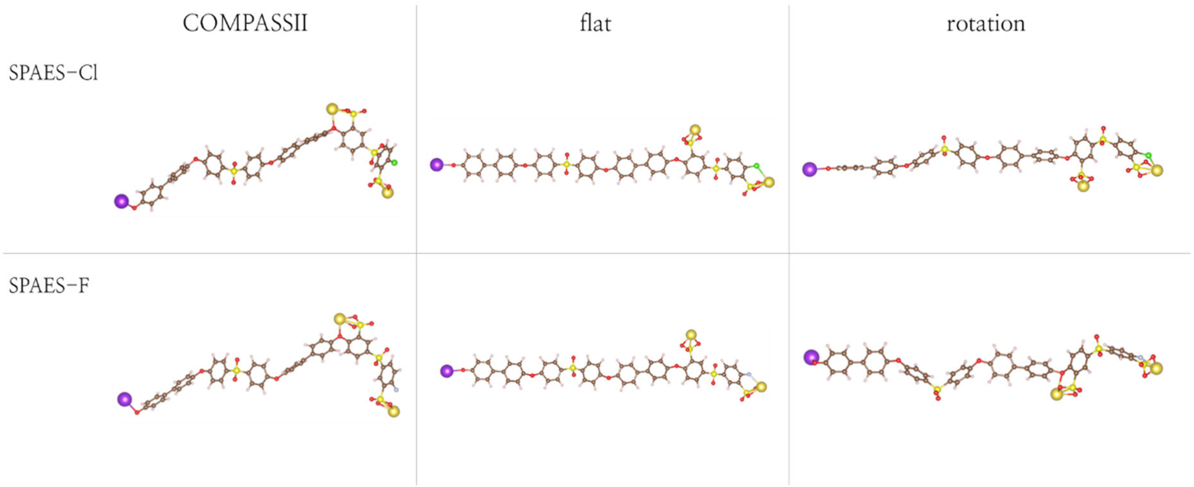

Figure S1. A repeating unit of SPAES calculated by applying COMPASSII, flat, and rotation respectively.

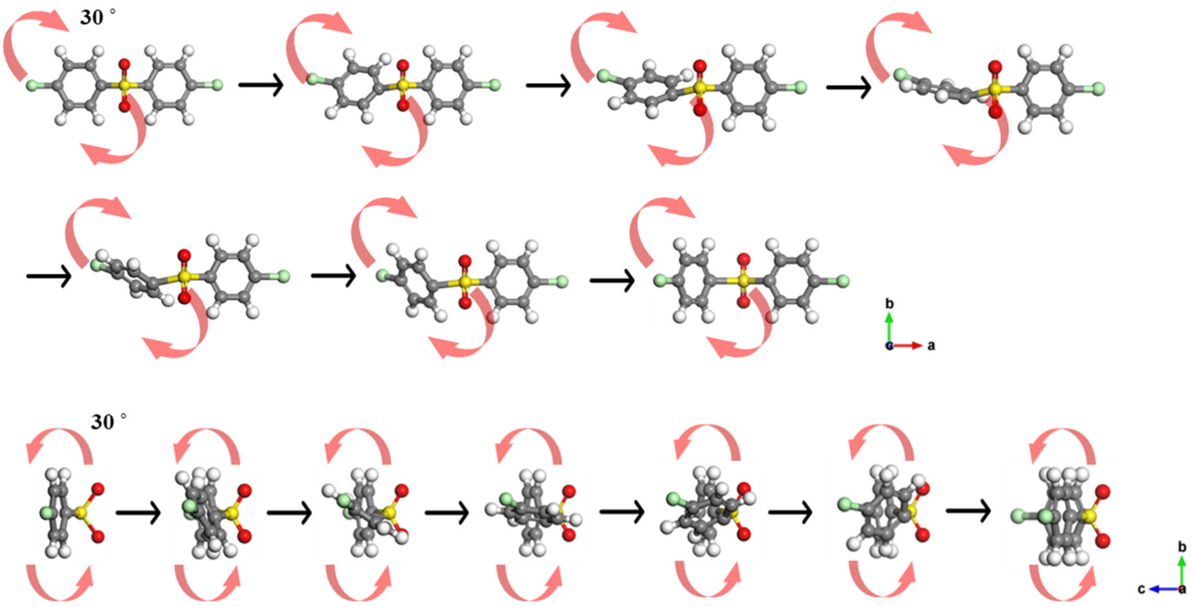

Figure S2. Illustration of rotating one side of a symmetric phenyl group.

* : Carbon, : Hydrogen, : Oxygen, : Sulfur, : Sodium : Chlorine, : Fluorine

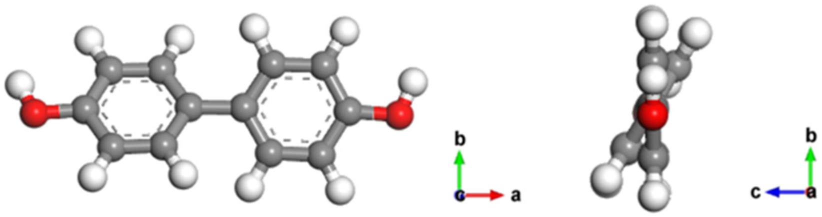

Figure S3. 4,4'-Dihydroxybiphenyl (BP, -OH form).

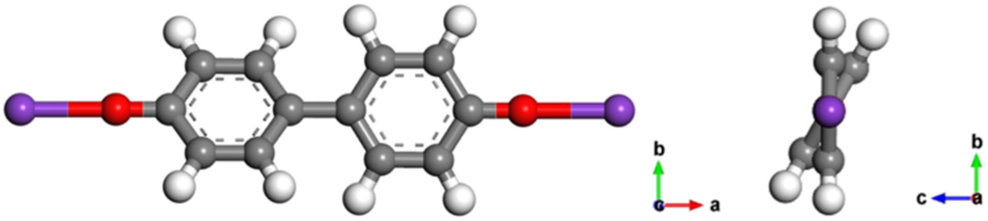

Figure S4. 4,4'-Dihydroxybiphenyl (BP, -OK form).

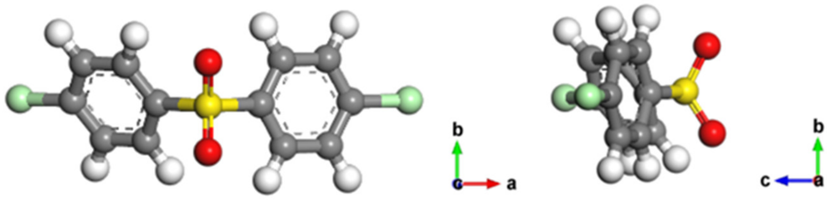

Figure S5. Bis(4-chlorophenyl) sulfone.

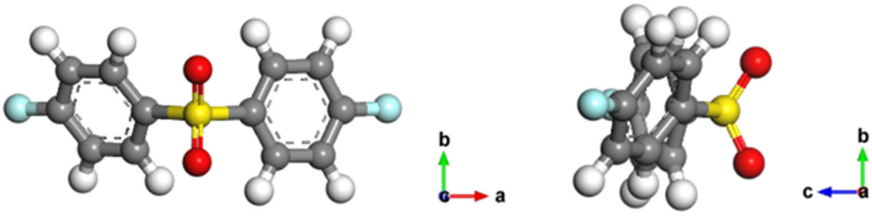

Figure S6. Bis(4-fluorophenyl) sulfone.

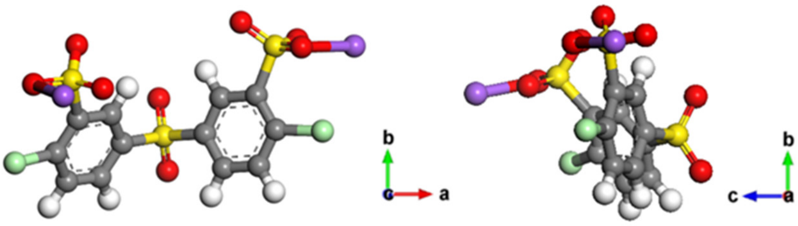

Figure S7. Bis(4-chlorophenyl-3-sulfophenyl) sulfone disodium salt.

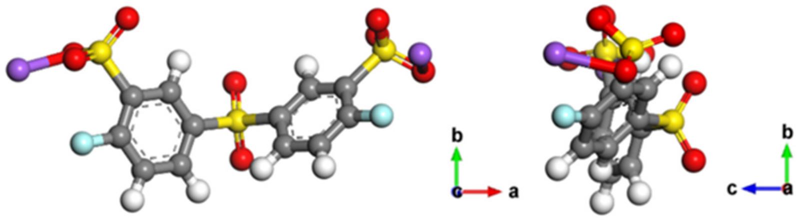

Figure S8. Bis(4-fluorophenyl-3-sulfophenyl) sulfone disodium salt.

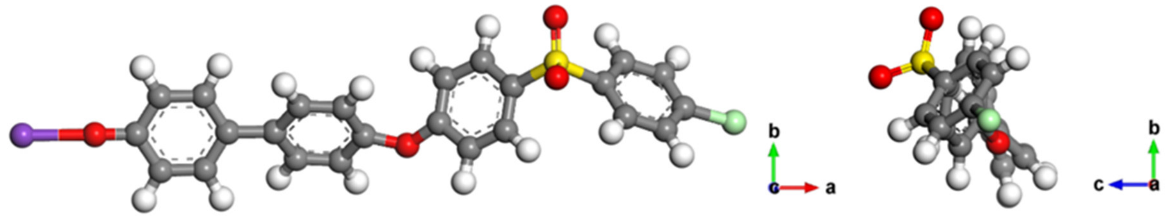

Figure S9. Bonding of 4,4'-Dihydroxybiphenyl and bis(4-chlorophenyl) sulfone.

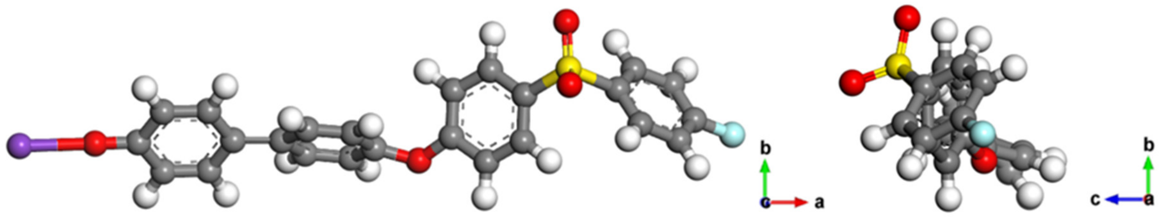

Figure S10. Bonding of 4,4'-Dihydroxybiphenyl and bis(4-fluorophenyl) sulfone.

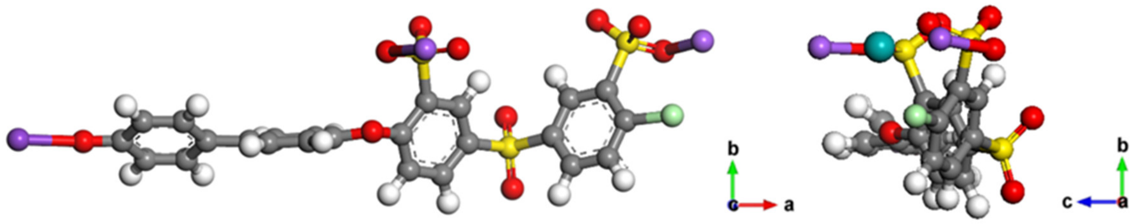

Figure S11. Bonding of 4,4'-Dihydroxybiphenyl and bis(4-chlorophenyl-3-sulfophenyl) sulfone disodium salt.

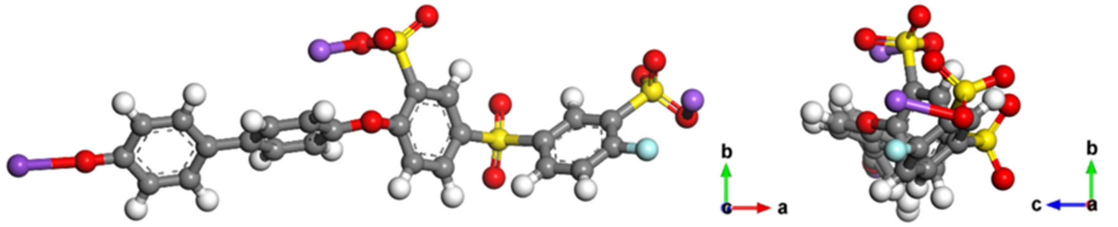

Figure S12. Bonding of 4,4'-Dihydroxybiphenyl and bis(4-fluorophenyl-3-sulfophenyl) sulfone disodium salt.

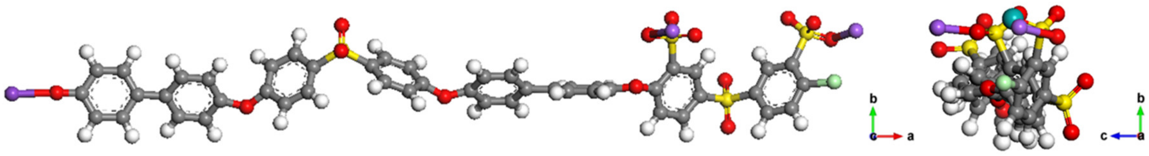

Figure S13. Sulfonated poly(arylene ether sulfone)(Cl form).

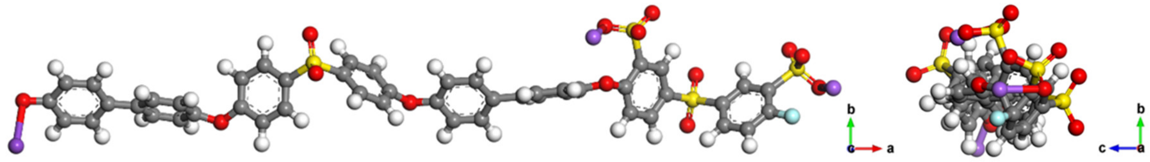

Figure S14. Sulfonated poly(arylene ether sulfone)(F form).

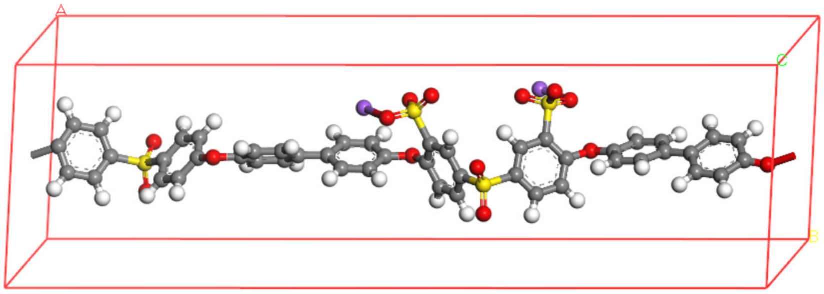

Figure S15. Sulfonated poly(arylene ether sulfone)(Cl form, periodic boundary condition).

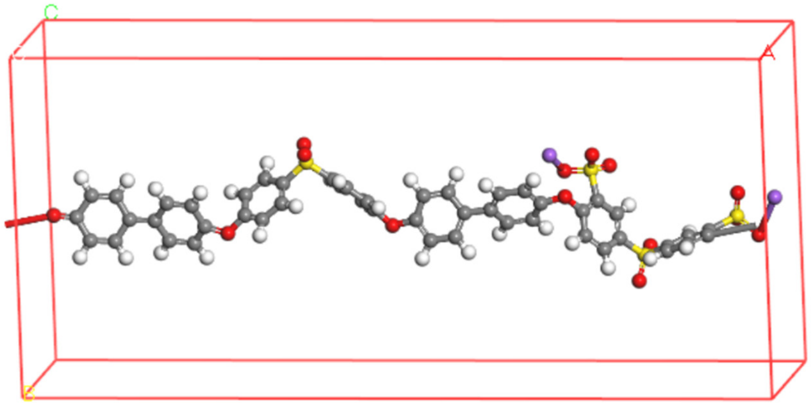

Figure S16. Sulfonated poly(arylene ether sulfone)(F form, periodic boundary condition).

S2. Gibbs free energy

The gaseous state of the salt is expressed as isolate and the solid state as bulk in **Figure S17**. One repeating unit of SPAES was composed of two 4,4'-dihydroxybiphenyl; BP, one biphenyl sulfone; BS, and one sulfonated biphenyl sulfone; SBS monomer. SPAES exist in the form of BP-BS-BP-SBS (**Figure S17a, S17b**) or BP-SBS-BP-BS (**Figure S17c, S17d**). Since sulfonated biphenyl sulfone has more heavy atoms (oxygen and sulfur) than biphenyl sulfone, the position of sulfonated biphenyl sulfone was expected to affect the polymerization process in which the polymer was formed. As a result of DFT calculation, it was showed that

the structure of BP-SBS-BP-BS was more stable in SPAES-Cl (**Figure S17d**), and the structure of BP-BS-BP-SBS in SPAES-F was more stable (**Figure S17b**).

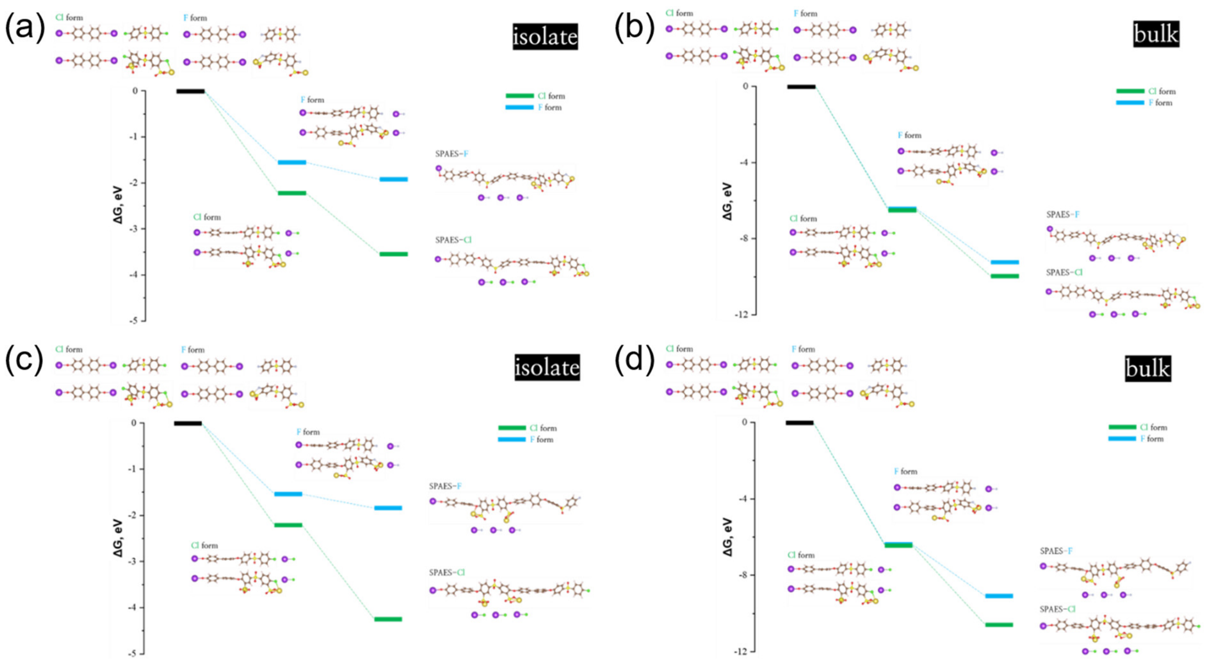

Figure S17. Gibbs free energy (ΔG) difference depending on the position of monomers and the state of salt.
